# The Effects of Acutely Administered 3,4-Methylenedioxymethamphetamine on Spontaneous Brain Function in Healthy Volunteers Measured with Arterial Spin Labeling and Blood Oxygen Level–Dependent Resting State Functional Connectivity

**DOI:** 10.1016/j.biopsych.2013.12.015

**Published:** 2015-10-15

**Authors:** Robin L. Carhart-Harris, Kevin Murphy, Robert Leech, David Erritzoe, Matthew B. Wall, Bart Ferguson, Luke T.J. Williams, Leor Roseman, Stefan Brugger, Ineke De Meer, Mark Tanner, Robin Tyacke, Kim Wolff, Ajun Sethi, Michael A.P. Bloomfield, Tim M. Williams, Mark Bolstridge, Lorna Stewart, Celia Morgan, Rexford D. Newbould, Amanda Feilding, H. Val Curran, David J. Nutt

**Affiliations:** a Centre for Neuropsychopharmacology (RLC-H, DE, LTJW, LR, SB, RT, AS, TMW, MB, DJN) and C3NL (RL), Division of Brain Sciences, Faculty of Medicine, London, London; b Division of Brain Sciences, Faculty of Medicine; c Psychiatric Imaging Group (MAPB), MRC Clinical Sciences Centre, Institute of Clinical Science, Imperial College London, London; d Institute of Neurology (MBW),; e Clinical Psychopharmacology Unit (BF, LS, CM, HVC), University College London, London; University College London, London; f Imanova (MBW, IDM, MT, RDN), Centre for Imaging Sciences, London; g The Beckley Foundation (AF), Beckley Park, Oxford; h Cardiff University Brain Research Imaging Centre (KM), School of Psychology, Cardiff University, Cardiff, London, United Kingdom; i School of Biomedical Sciences (KW), Kings College London, London, United Kingdom

**Keywords:** Amygdala, 5-HT, fMRI, Hippocampus, MDMA, PTSD, Serotonin

## Abstract

**Background:**

The compound 3,4-methylenedioxymethamphetamine (MDMA) is a potent monoamine releaser that produces an acute euphoria in most individuals.

**Methods:**

In a double-blind, placebo-controlled, balanced-order study, MDMA was orally administered to 25 physically and mentally healthy individuals. Arterial spin labeling and seed-based resting state functional connectivity (RSFC) were used to produce spatial maps displaying changes in cerebral blood flow (CBF) and RSFC after MDMA administration. Participants underwent two arterial spin labeling and two blood oxygen level–dependent scans in a 90-minute scan session; MDMA and placebo study days were separated by 1 week.

**Results:**

Marked increases in positive mood were produced by MDMA. Decreased CBF only was observed after MDMA, and this was localized to the right medial temporal lobe (MTL), thalamus, inferior visual cortex, and the somatosensory cortex. Decreased CBF in the right amygdala and hippocampus correlated with ratings of the intensity of global subjective effects of MDMA. The RSFC results complemented the CBF results, with decreases in RSFC between midline cortical regions, the medial prefrontal cortex, and MTL regions, and increases between the amygdala and hippocampus. There were trend-level correlations between these effects and ratings of intense and positive subjective effects.

**Conclusions:**

The MTLs appear to be specifically implicated in the mechanism of action of MDMA, but further work is required to elucidate how the drug’s characteristic subjective effects arise from its modulation of spontaneous brain activity.

The compound 3,4-methylenedioxymethamphetamine (MDMA) releases serotonin (5-hydroxytryptamine [5-HT]), dopamine, and norepinephrine (1). It is also a popular recreational drug that is valued by users because of its acute prosocial and euphoretic properties (2). Although MDMA has been administered in human research on numerous occasions (3–5), few studies have investigated its acute effects on brain function using functional magnetic resonance imaging (fMRI) (6–8) or other neuroimaging modalities (9–11).

The compound MDMA has a relatively unique profile of subjective effects, described as a hybrid between a stimulant and psychedelic (12). It acts at dopamine, norepinephrine, and 5-HT transporters to inhibit reuptake and stimulate release; however, the greater action of MDMA at the serotonin transporter differentiates it from most other stimulants (13) and accounts for much, but not all, of its euphoretic effects (14,15). Although the pharmacology of MDMA is reasonably well understood, little is known about its effects on global brain function. More recently, MDMA has been investigated as a potential adjunct to psychotherapy in the treatment of posttraumatic stress disorder (PTSD), with positive, albeit preliminary, outcomes (16,17).

Despite significant developments in resting state fMRI in recent years (18), there have been no resting state fMRI studies on the acute effects of MDMA. In the present study, we combined arterial spin labeling (ASL) and resting state functional connectivity (RSFC) to address this knowledge gap. The magnetic resonance imaging technique ASL provides a quantitative measure of cerebral blood flow (CBF) or perfusion (19), and RSFC measures functional coupling between spatially distributed brain regions via spontaneous fluctuations in the blood oxygen level–dependent (BOLD) signal (20). Combining these complementary techniques can yield important new information on how a drug alters brain activity to produce its characteristic subjective effects (21). Given the recognized acute prosocial and positive mood effects of MDMA (6,22), we predicted changes in CBF and RSFC in brain systems implicated in social and affective processing—limbic structures and the medial prefrontal cortex (mPFC) (23,24). On this basis, three regions (i.e., ventromedial prefrontal cortex [vmPFC], bilateral hippocampi, and amygdalae) were selected for seed-based RSFC analyses (20).

Supporting the importance of this research is: 1) the relative dearth of human functional neuroimaging data on what is one of the most popular drugs of potential misuse (25); 2) the ability of MDMA to produce an acute state of euphoria and the poor understanding of the neural underpinnings of such states (26); 3) the ability of MDMA to produce marked 5-HT release (13), supporting its utility in serotoninergic challenge (27); and 4) preliminary evidence for the potential of MDMA as a therapeutic agent (17).

## Methods and Materials

Supplement 1 contains the complete Methods and Materials section.

### Design

**Figure 1:**
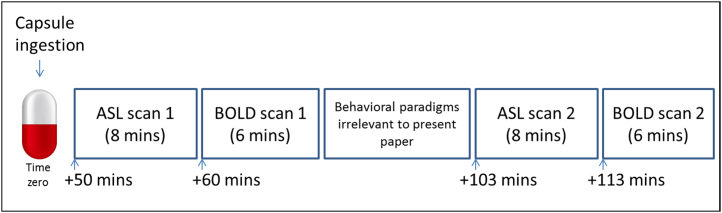
Schematic showing scanning protocol. Placebo (vitamin C) or 3,4-methylenedioxymethamphetamine (MDMA) hydrochloride (100 mg) was ingested at time zero, and the first arterial spin labeling scan performed 50 min later. This was a repeated measures design; the two scans (placebo and MDMA) were performed 1 week apart, and the scan order was counterbalanced so that half of the volunteers received MDMA for the first scan, and half received MDMA for the second scan. ASL, arterial spin labeling; BOLD, blood oxygen level–dependent.

This was a within-subjects, double-blind, randomized, placebo-controlled study. Participants were scanned twice, 7 days apart—once after MDMA and once after placebo. A schematic of the scanning protocol is shown in Figure 1. The study was approved by the National Research Ethics Service West London Research Ethics Committee, Joint Compliance and Research Office of Imperial College London, Research Ethics Committee of Imperial College London, Head of the Department of Medicine of Imperial College London, Imanova Centre for Imaging Science, and Faculty of Medicine of Imperial College London. The study was conducted in accordance with Good Clinical Practice guidelines. A Home Office Licence was obtained for the storage and handling of a Schedule 1 drug. Imperial College London sponsored the research.

### Participants

The study included 25 healthy participants (mean age, 34 ± 11 years; 7 females) with at least one previous experience with MDMA. None of the participants had used MDMA for at least 7 days or other drugs for at least 48 hours, which was confirmed by a urine screen. An alcohol breathalyzer test confirmed that none of the participants had recently consumed alcohol. Participants had used MDMA an average of 35 ± 51 times before (range, 1–200 times), and the mean time since last use was 1400 ± 2351 days (range, 7–7300 days). Participants were screened for good physical and mental health, and magnetic resonance imaging compatibility. Screening involved routine blood tests, electrocardiogram, heart rate, blood pressure, and a brief neurologic examination. The Mini International Neuropsychiatric Interview version 5 (MINI-5) was performed by an experienced psychiatrist to assess mental health. All subjects were deemed physically and mentally healthy, and none had any history of drug or alcohol dependence or diagnosed psychiatric disorder. Participants had mean Beck Depression Inventory scores of 3.9 ± 4.8 (range, 0–18) and Spielberger State-Trait Anxiety Inventory scores of 31.7 ± 5.9 (range, 20–46).

## Results

### Basic Subjective and Physiologic Effects

**Figure 2:**
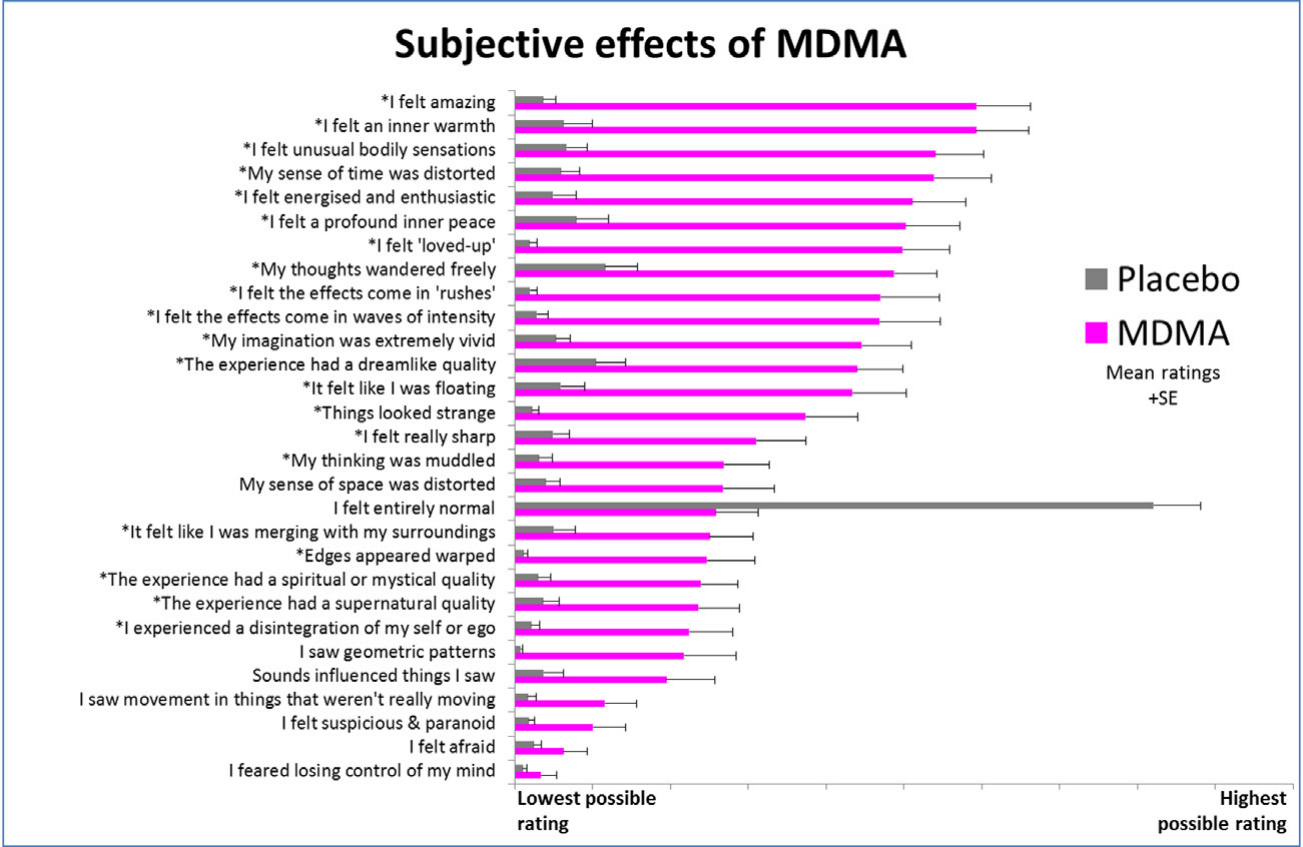
Subjective effects of 3,4-methylenedioxymethamphetamine (MDMA). There were 29 items rated 4 hours after drug administration. Participants were instructed to complete the items with reference to the peak drug effects (where applicable). The items marked with an asterisk were rated significantly higher after MDMA than placebo (*p* < .001, Bonferroni correction for multiple comparisons). The mean ratings for 25 participants are shown plus the positive standard errors from the mean (SE).

The intensity of the subjective effects of MDMA was variable across subjects. Five subjects failed to notice any subjective effects during the scanning period, whereas three gave maximal ratings, indicating “extremely intense” effects. Peak drug effects were reported 100 min after ingestion of MDMA (the intensity was rated at 52 ± 32%; range, 0%–100%; 0% = no effects and 100% = extremely intense effects) coinciding with the beginning of the second ASL scan (103 min after capsule ingestion). However, the average intensity remained relatively consistent throughout the scanning period (i.e., intensity was rated at 44 ± 35% at the end of the first ASL scan and 43 ± 32% at the end of the second BOLD scan). Most volunteers reported positive mood effects after MDMA, and items referring to aspects of positive mood were among the highest scored (e.g., the item “I felt amazing” was the highest rated item after MDMA administration) (Figure 2).

### Mean Plasma Concentration of MDMA

Biochip Array Technology (Randox Laboratories Ltd., Co., Antrim, United Kingdom) was used to detect MDMA from plasma samples obtained shortly after each participant’s MDMA scanning session (i.e., 2 hours after capsule ingestion). The mean concentration of MDMA was 214 ± 66 ng/mL.

### ASL Results

**Figure 3:**
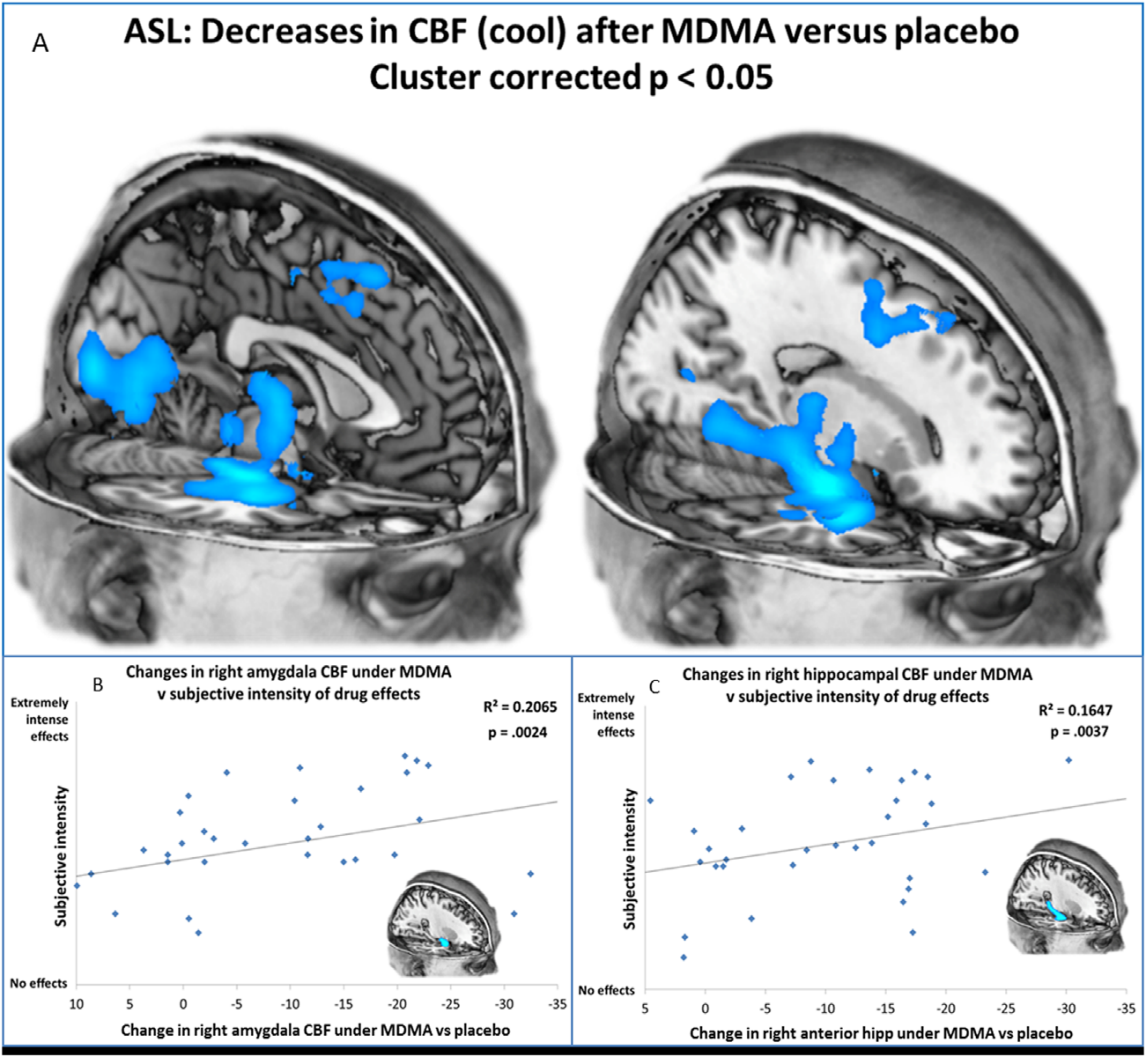
Decreases in cerebral blood flow (CBF) after administration of 3,4-methylenedioxymethamphetamine (MDMA). **(A)** Regions of significantly less CBF after MDMA administration (scans 1 and 2) vs. placebo (scans 1 and 2) are displayed. These images are cluster-corrected giving a whole-brain corrected statistical threshold of *p* < .05. See Supplement 1 for additional slices. **(B,C)** Decreased right amygdala and hippocampal CBF predicts intense subjective effects after MDMA. Values on the x-axis are ratings from the first and second arterial spin labeling scans after MDMA administration. A corrected *p* value of < .005 was used. The decreases in CBF after MDMA administration versus placebo increase in magnitude from left to right. The greater the decreases in CBF in the amygdalae and hippocampi after MDMA administration, the more intense were the drug’s subjective effects. ASL, arterial spin labeling; hipp, hippocampus.

Subtracting the two ASL scans after MDMA administration from the two ASL scans after placebo revealed robust decreases in CBF after MDMA. The images shown in Figure 3A were produced using cluster-correction (2590 voxels) to adjust for multiple comparisons and a whole-brain corrected statistical threshold of *p* < .05. At this threshold, decreases in CBF only were observed, and these were localized to the regions shown in Figure 3A. Increases in CBF could be observed only at an unacceptable statistical threshold of *p*_uncorrected_ < .3. For a more comprehensive display of the regional decreases in CBF after MDMA, see Supplement 1.

When contrasts were split so that the effect of MDMA in the first and second ASL scans could be observed separately, consistent maps were revealed, with decreases in CBF only after MDMA. The decreases were slightly more marked and of a greater spatial extent in the second ASL scan than the first (Supplement 1).

### Correlations between CBF Effects and Subjective Ratings

Regions showing the most marked reductions in CBF after MDMA administration included the visual cortex, thalamus, somatosensory cortex, right hippocampus, and right amygdala. Correlational analyses were restricted to these regions of interest. Masks were derived from an anatomic atlas, and CBF changes in the relevant regions were correlated with self-ratings of the intensity of the subjective effects of MDMA. Significant positive correlations were observed between the magnitude of the CBF decreases in the right amygdala (*p* = .002) and right hippocampus (*p* = .004) after MDMA administration and the subjective intensity of the drug effects (Figure 3B,C). Correcting for multiple comparisons gave a revised statistical threshold of *p* < .005 (.05/10), and these correlations survived this threshold. Because the amygdala and hippocampus are limbic structures known to be involved in affective processing, we also examined correlations between the CBF changes and ratings of increased positive affect after MDMA administration, and although correlations were in the predicted direction, no significant relationships were found.

### RSFC Results

When vmPFC RSFC after MDMA administration was contrasted against vmPFC RSFC after placebo administration, significant increases (yellow-orange color) and decreases (blue color) were observed (cluster-corrected, *z* = 2.3, *p* < .05; this threshold was used for all of the RSFC analyses). Increases in vmPFC RSFC were observed in visual cortex, both medially and laterally (left and right hemispheres). Decreases were found in the midbrain (including voxels in the vicinity of the dorsal raphe nuclei), thalamus, amygdala, and posterior cingulate cortex (PCC).

When hippocampal RSFC after MDMA administration was contrasted against hippocampal RSFC after placebo administration, significant increases in RSFC were observed in the dorsal ACC, right amygdala, and right middle frontal gyrus. Decreases were found in the mPFC, left posterior parahippocampal/fusiform gyrus, and left temporal cortex.

When amygdala RSFC after MDMA administration was contrasted against amygdala RSFC after placebo administration, significant increases in RSFC were observed in the brainstem and bilaterally in the anterior parahippocampal gyrus. Decreases in RSFC were found in the cerebellum, left temporal cortex, medial orbitofrontal cortex, and subgenual cingulate cortex. For images of positive RSFC to the regions of interest during baseline conditions, see Supplement 1.

### Relationship between Changes in RSFC and Subjective Effects of MDMA

Six correlations were tested, giving a revised statistical threshold of *p* < .008 (.05/6). Specifically, between-condition differences in vmPFC-PCC, hippocampal-vmPFC, and amygdala-hippocampal RSFC versus ratings of the intensity of the global subjective effects of MDMA and ratings of positive mood were tested. There was a trend toward decreased vmPFC-PCC RSFC after MDMA administration correlating with intense (*r* = .13, *p* = .218) and positive (*r* = .36, *p* = .038) subjective effects, but neither correlation was significant. Similarly, there was a trend toward decreased hippocampal-vmPFC RSFC after MDMA administration correlating with intense (*r* = .32, *p* = .026) and positive (*r* = .3, *p* = .073) effects, but neither correlation was significant. Finally, there was a trend toward increased amygdala-hippocampal RSFC correlating with intense (*r* = .382, *p* = .01) and positive (*r* = .159, *p* = .225) subjective effects, but these correlations were not significant when corrected for multiple testing.

### Addressing Between-Condition Differences in Motion as a Potential Confounder

All available methods were employed to control for subjective motion in the RSFC analyses (e.g., motion parameter time courses and outlier volumes were included as confounder variables in the first-level general linear models). In addition, between-condition motion in the resting state BOLD scans was formally compared. There was significantly more movement in the MDMA than placebo scans (*p* = .003); however, the magnitude of this difference was so small as to be functionally insignificant (i.e., the mean relative movement per volume in the RSFC scans was .072 ± .04 mm after placebo administration and 0.099 ± .08 mm after MDMA administration. Mean motion failed to explain any of the variance in the main RSFC outcomes when tested in post hoc regression analyses containing the between-condition differences in RSFC as the dependent variable and between-condition differences in motion as a single explanatory variable.

### Addressing Between-Subject Differences in Drug Use as a Potential Confounder

Because there was a large variability in previous drug use among the study sample, additional regression analyses were run to test for relationships between drug use and between-condition differences in RSFC. Specifically, using the same approach outlined previously, between-condition differences in RSFC were entered as the dependent variable, and previous MDMA exposure, recency of MDMA exposure, weekly alcohol use, and lifetime cannabis use were entered separately as single explanatory variables. Between-subject variance in drug use failed to explain significantly any of the between-condition RSFC outcomes.

## Discussion

This is the first resting state fMRI study on the acute effects of MDMA on spontaneous brain function. Decreased CBF was seen in the amygdala and hippocampus, and this correlated with the intensity of the drug’s effects. Decreases in vmPFC-MTL and vmPFC-PCC RSFC and increase in amygdala-hippocampal RSFC were also observed, and there were trend-level correlations between these effects and the intensity and positive mood effects of MDMA.

The CBF decreases after MDMA were localized to the subcalcarine visual cortex, pre–supplementary motor area, somatosensory cortex, superior frontal gyrus, midbrain and brainstem, thalamus, hippocampus and parahippocampus, and amygdala. The 5-HT_1B_ receptor is especially densely expressed in the subcalcarine domain of the visual cortex (28), which is precisely where the CBF decreases in the visual cortex were observed. It is natural to infer that endogenous 5-HT released by MDMA may have stimulated this particular 5-HT receptor in this particular region to produce the observed decreases in CBF. Supporting the role of 5-HT in mediating this and the other main effects, MDMA produces a 5-fold greater increase in synaptic 5-HT than dopamine (13), and dopamine and norepinephrine receptors are not densely expressed in the visual cortex.

The decreases in CBF in the MTLs were one of the most intriguing results of this study, particularly because the magnitude of these decreases correlated positively with ratings of the drug’s global subjective effects, even after correcting for multiple comparisons (Figure 3B,C). The MTL structures receive an especially dense serotoninergic innervation (29), and 5-HT is found in higher concentrations in the hippocampus than dopamine and norepinephrine (30). The hippocampus (31) and amygdala (32) express postsynaptic 5-HT_1A_ receptors in high concentrations, endogenous 5-HT has a relatively high affinity for 5-HT_1A_ receptors (27), and the effects of 5-HT stimulation of 5-HT_1A_ receptors are hyperpolarization and a decrease in cell firing rate (33). Other 5-HT receptors are expressed in the hippocampus, amygdala, and parahippocampus (e.g., the 5-HT_7_ receptor and 5-HT_2A_ receptor (34,35)) but to a far lesser extent than the 5-HT_1A_ receptor (36). It is reasonable to infer that the marked decreases in CBF in the MTLs were caused by an effect of 5-HT on inhibitory postsynaptic 5-HT_1A_ receptors.

Elevated limbic activity is a reliable characteristic of anxiety states (37). Serotoninergic medications with anxiolytic properties, such as selective serotonin reuptake inhibitors and 5-HT_1A_ receptor agonist buspirone, are thought to elicit their therapeutic action via stimulation of inhibitory postsynaptic 5-HT_1A_ receptors, normalizing limbic activity (38). Acutely administered MDMA does not appear to have typical anxiolytic properties in either animals or humans (17,39); however, the subjective ratings displayed in Figure 2 clearly demonstrate an increase in positive mood under the influence of the drug (albeit without any effect on negative mood—which was already low). Prosocial behaviors have previously been observed after MDMA administration (2,22,40), and MDMA-induced prosocial behavior in rats was reduced after pretreatment with a selective 5-HT_1A_ receptor antagonist (41). Given the potent serotonin releasing properties of MDMA (1,13), it can be inferred that the reduced MTL CBF observed here was mediated by 5-HT_1A_ receptor stimulation and is related, at least in part, to the drug’s positive mood effects. However, contradicting the role of 5-HT_1A_ receptors in the mechanism of action of MDMA is the finding that pretreatment with pindolol does not significantly attenuate the drug’s subjective effects (14,42); pindolol is a partial agonist that may not provide effective blockade of postsynaptic 5-HT_1A_ receptors (43,44). Pretreatment studies with potent and selective antagonists are required to elucidate the specific receptor subtypes mediating the decreases in CBF.

Similar to the ASL outcomes, the RSFC analyses also yielded robust results. For example, the decreases in vmPFC-PCC coupling after MDMA administration are of interest given more recent evidence that increased vmPFC-PCC coupling is positively associated with rumination in depression (45). On this basis, we had predicted that the decreases in vmPFC-PCC RSFC would correlate with the drug’s positive mood effects, but although there was a trend in this direction, it was not significant after correction or multiple comparisons. Decreased vmPFC-PCC RSFC has also been found with psilocybin (21), a nonselective 5-HT_2A_ receptor agonist with potent consciousness-altering properties. Psilocybin produces an unconstrained style of cognition that is the inverse of the constrained, ruminative style of thinking that is characteristic of depression. Participants described a similar liberation of cognition and imagination after MDMA administration (Figure 2), and vmPFC-PCC coupling was decreased after administration of the drug. In future research with MDMA, it would be interesting to incorporate pretreatment with a selective 5-HT_2A_ receptor antagonist to test the involvement of this specific receptor in mediating the drug’s effects. The 5-HT_2A_ receptor is highly expressed in both the mPFC and PCC (46), and 5-HT_2A_ receptor blockade was found to significantly attenuate the positive mood effects of both MDMA (14) and psilocybin (47).

**Figure 4:**
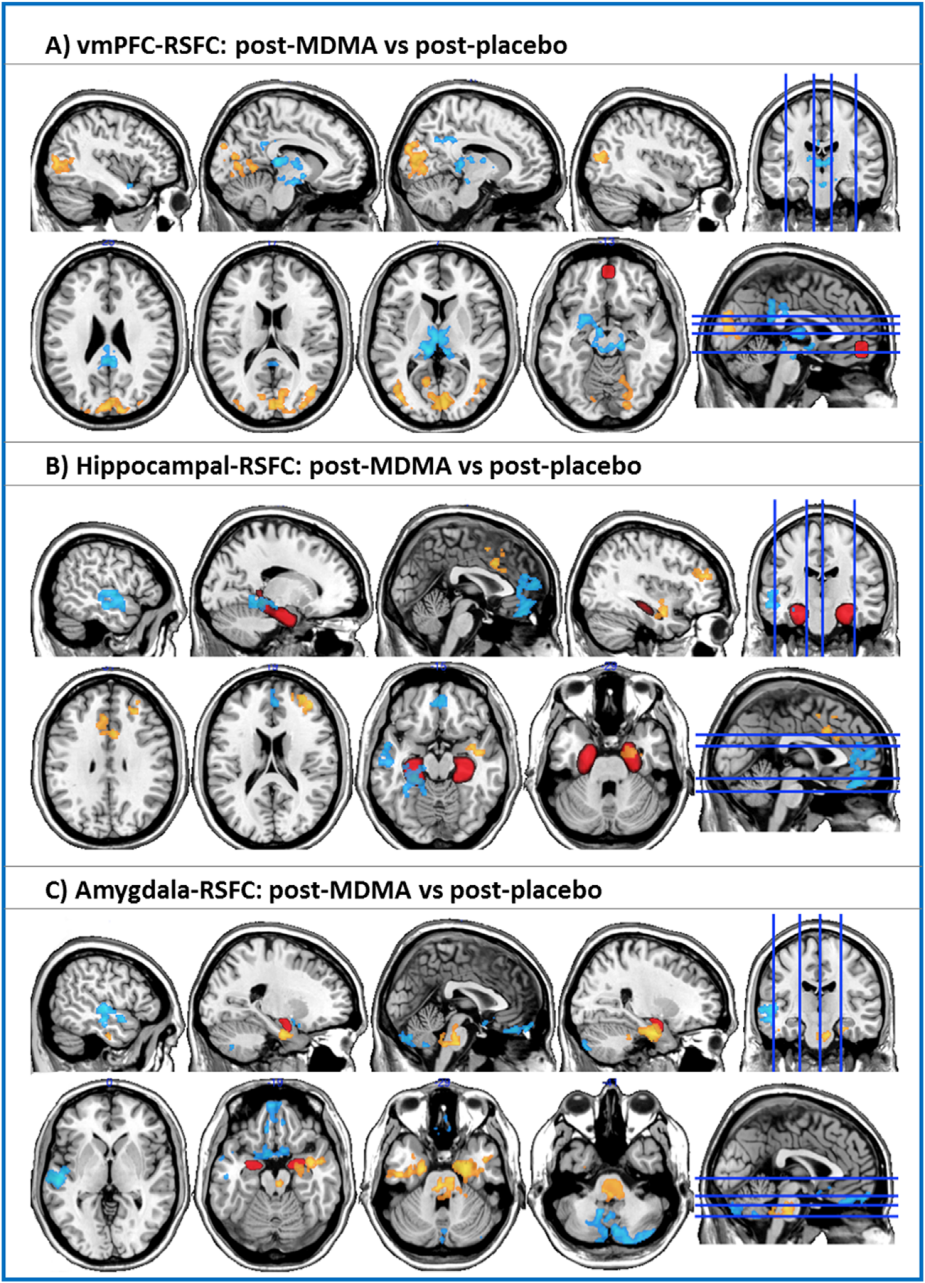
Effect of 3,4-methylenedioxymethamphetamine (MDMA) on resting state functional connectivity (RSFC). **(A)** Changes in ventromedial prefrontal cortex (vmPFC) RSFC. **(B)** Changes in hippocampal RSFC after MDMA administration. **(C)** Changes in amygdala RSFC after MDMA administration. Increases in RSFC are shown in yellow-orange, and decreases in RSFC are shown in blue. All seeds are shown in red. The blue lines on the axial and sagittal slices on the far right indicate the planar position of the preceding slices. All images were cluster-corrected, *z* = 2.3, *p* < .05.

Regarding other circuitry implicated in the action of MDMA, decreased mPFC-hippocampal RSFC was observed (Figure 4). The uncinate fasciculus connects the vmPFC and MTL structures (48), and other indirect connections (e.g., via the retrosplenial cortex and ventral PCC) likely account for the substantial baseline functional connectivity seen between these regions (Supplement 1). Research in rodents has shown that the mPFC exerts a top-down inhibitory influence on limbic activity (49), often observed in the context of emotional control (50). These regions have also been implicated in the pathophysiology of PTSD. For example, patients with pronounced dissociative symptoms exhibit elevated mPFC and reduced MTL responses to trauma-related cues (51) presumably as a result of an exaggerated influence of the mPFC on the MTLs (52). More recently, MDMA has begun to be formally investigated as an adjunct to psychotherapy for PTSD (16,17). It is claimed that MDMA aids patients’ ability to cope with the distress of recollecting their trauma when required to do so in psychotherapy (16). Similar to limbic hyperactivity, increased coupling between the mPFC and hippocampus is a marker of anxiety states and appears to be modulated by the 5-HT_1A_ receptor (53). There was a trend-level positive correlation between the magnitude of the decreases in mPFC-hippocampal coupling after MDMA administration and ratings of positive mood and intensity of the drug’s global effects. Further work is required to investigate the hypothesis that the positive mood effects of MDMA are mediated, at least in part, by decreased mPFC-hippocampal and mPFC-PCC coupling.

Although many aspects of the RSFC results are interesting, we have focused on the effects that were especially marked and are related to relevant previous work. The final effect given special attention is the increased coupling between the amygdala and the hippocampus after MDMA administration. The magnitude of the increases in amygdala-hippocampal RSFC correlated at a near significant level with ratings of the intensity of the global effects of MDMA. Decreased amygdala-hippocampal RSFC has been found in patients with PTSD relative to combat veterans without PTSD (54). The authors of the study speculated that the decrease in amygdala-hippocampal RSFC may relate to an impaired ability to contextualize affective information in PTSD. It is intriguing that MDMA had an inverse effect on amygdala-hippocampal RSFC in the present study, increasing it in a manner that correlated with the drug’s global subjective effects (albeit at a trend level). Further work is required test both the safety and efficacy of MDMA in PTSD and the specific mechanisms by which it may be effective. There is only preliminary evidence from a single published pilot study to support the therapeutic potential of MDMA in the treatment of PTSD (17). However, the results of the present study indicate that the MTLs may be specifically implicated in any potential therapeutic action of the drug.

There have been no previous resting state fMRI studies on MDMA, but a steady-state positron emission tomography study measured CBF after administration of 119 mg/70 kg MDMA in 16 healthy volunteers (9). Because the experimental conditions differed from the conditions of the present study (e.g., participants performed a low-level cognitive task during many of the scans), it is difficult to compare the study outcomes. Some decreases in CBF were observed in the thalamus, amygdala, and somatosensory cortex in the positron emission tomography study, but increases in CBF (in the orbitofrontal cortex, visual cortex, and cerebellum) were also observed. In another positron emission tomography study of a proserotoninergic agent, intravenous fenfluramine was administered during steady-state cognition, and increased frontal cortical and decreased thalamic CBF was observed (55).

The present study is the largest and most advanced acute MDMA human imaging study to date; however, it has some important limitations. We did not incorporate retrospective correction of physiological motion effects to correct for the physiologic variance (56). However, this process had a negligible effect on the results when previously applied to psilocybin fMRI data (21). Similarly, the breath-hold paradigm incorporated into the psilocybin fMRI design to test for drug-vascular interactions did not reveal any modulatory influence with this serotoninergic agent. Nevertheless, given the hemodynamic nature of the ASL and BOLD signals, it remains plausible that some of the observed effects were caused by a direct vascular action of MDMA or (released) serotonin, and the design would have benefited from the inclusion of RETROICOR or a breath-hold paradigm, or both. However, contradicting a direct vascular action of the drug, the observed CBF and RSFC effects were localized to functionally meaningful brain regions (e.g., the MTLs) rather than being global in extent or proximal to regions with a high vascular input, and the decreases in MTL CBF correlated with global subjective effects of MDMA.

Five of the 25 participants in this study were filmed as part of a television documentary on the effects of MDMA. These 5 participants were not filmed during scanning and completed the study protocol in the same way as the other 20 participants. However, to address concerns about filming being a potential confounding variable, we reanalyzed the ASL data after removing the five filmed participants, and the main effects of MDMA were unchanged (Supplement 1).

A definite limitation of this study was the lack of a pharmacologic pretreatment component. Several pretreatment studies with MDMA in humans have now been published (14,42,57–66), and an advanced design would have included an antagonist pretreatment component to elucidate the pharmacologic mechanism underlying the fMRI-measured effects of MDMA.

The hypothesis-driven nature of our seed-based RSFC analyses could also be questioned. Seed-based RSFC requires prior selection of specific seeds, and if prior hypotheses about the functional importance of the chosen seeds are tenuous or lacking, this selection process can seem arbitrary. However, the selection of MTL seeds and the vmPFC in the present study can be justified given their association with social and affective processing (67,68) and the recognized modulatory influence of MDMA on these functions (6,22,69). Nonetheless, other regions of interest could have been selected if informed by specific prior hypotheses. In contrast to seed-based RSFC, independent components analysis is a data-driven technique that could have been applied to the present data to identify resting state networks, which could have been scrutinized in between-condition analyses, either looking at between-network RSFC between conditions or differences in RSFC within the independent components analysis–defined networks. Relevant independent components analysis–based analyses are the focus of a separate publication.

It would be misleading to infer that changes in RSFC between a seed and other regions in the brain apply exclusively to the selected seed. Indeed, the same between-condition differences in RSFC may be shared by multiple regions. Related to this, RSFC analyses do not provide information on the causal source of changes in RSFC, and to address such questions one needs to consider exploring effective-connectivity measures (70).

Another potential limitation of the study was the inclusion of behavioral paradigms between the first and second pair of resting state scans. It is possible that these had carry-over effects on the CBF and RSFC outcomes of the second pair of scans. However, this possibility seems unlikely given that the outcomes of the second ASL and BOLD resting state scans were consistent with those of the first pair (Supplement 1).

Finally, the effectiveness of the blinding procedure is compromised when studying the acute effects of a relatively potent psychoactive drug such as MDMA. Participants correctly identified when they had received MDMA or placebo in 45 of the 50 study days, and the research team predicted correctly in 48 of the study days. It is difficult to circumvent this issue. A very low dose of MDMA or another stimulant such as amphetamine could have been added as a control condition. However, a drug-free baseline is required to properly determine the effects of an experimental compound. With this said however, the ineffectiveness of blinding needs to be highlighted as a study limitation.

In conclusion, this is the first study to have used resting state fMRI to address the question of how MDMA works on the human brain to produce its characteristic subjective effects. The results revealed decreased CBF in MTL regions, decreased RSFC between the vmPFC and PCC, decreased mPFC-hippocampus RSFC, and increased amygdala-hippocampus RSFC. Taken together, the MTL regions appear to be specifically implicated in the mechanism of action of MDMA. However, these results should be seen as informative rather than confirmatory, and further research is required to elucidate the precise mechanisms by which the characteristic subjective effects of MDMA arise from its modulation of brain activity.
