# Supplementary material for: The Effects of Acutely Administered 3,4-Methylenedioxymethamphetamine on Spontaneous Brain Function in Healthy Volunteers Measured with Arterial Spin Labeling and Blood Oxygen Level–Dependent Resting State Functional Connectivity
Source: Biol Psychiatry. 2015 Oct 15;78(8):554–62. doi: 10.1016/j.biopsych.2013.12.015 (PMC4578244; doi:10.1016/j.biopsych.2013.12.015)
Supplement: Supplementary file 1 — Supplementary Material [file mmc1.pdf]

The Effects of Acutely Administered 3,4-Methylenedioxymethamphetamine on Spontaneous Brain Function in Healthy Volunteers Measured with Arterial Spin Labelling and Blood Oxygen Level-Dependent Resting-State Functional Connectivity

Supplemental Information

Supplemental Methods & Materials

Participants

All subjects were deemed physically and mentally healthy and none had any history of drug or alcohol dependence or diagnosed psychiatric disorder. Other drug use parameters were as follows (values are means ± SD (range)): Alcohol weekly units 12.8 ± 10.2 (0-35), daily cigarettes 1.7 ± 4.6 (0-20), cannabis lifetime uses 267.4 ± 323 (0-1000+), LSD lifetime uses 27.3 ± 100 (0-500), psilocybin lifetime uses 9.5 ± 20 (0-100), ketamine lifetime uses 21.6 ± 46.7 (0-200), mephedrone lifetime uses 3.6 ± 7.4 (0-30), amphetamine lifetime uses 17.8 ± 36.9 (0-150), cocaine lifetime uses 49.6 ± 152 (0-750).

Scanning Parameters

MR images were acquired on a 3T Siemens Tim Trio (Siemens Healthcare, Erlangen, Germany) using a 32-channel phased array head coil. Anatomical reference images were acquired using the ADNI-GO recommended MPRAGE parameters (1 mm isotropic voxels, TR = 2300 ms, TE = 2.98 ms, 160 sagittal slices, 256 x 256 in-plane resolution, flip angle = 9 degrees, bandwidth = 240 Hz/pixel, GRAPPA acceleration = 2). T2*-weighted echo-planar images (EPI) were acquired for the resting state functional scan using 3 mm isotropic voxels in a 192 mm in-plane FOV, TR = 2 s, echo time = 31 ms, 80 degree flip angle, 36 axial slices in each TR, bandwidth = 2298 Hz/pixel, and a GRAPPA acceleration of 2. One hundred eighty

volumes were acquired during the functional imaging paradigm which took 6 minutes to complete and there were 2 of these resting state scans.

Arterial Spin Labeling (ASL) Parameters

ASL flow maps were acquired using the Q2TIPS pulsed ASL preparation with a 2D EPI readout (1). At each inflow time, 16 5-mm slices were imaged in a 240 mm FOV, 14 ms TE, 64 x 64 matrix, 2500 ms TR, and 2232 Hz/pixel bandwidth, giving 3.8 mm in-plane resolution. Thirteen alternating tag-control pairs plus an initial M0 calibration volume were acquired in one minute and 15 seconds for each inflow time. Seven inflow times from 750 ms to 1650 ms in steps of 150 ms were taken.

Drug and Dosing Parameters

There were 2 ASL and 2 blood oxygen level-dependent (BOLD) resting-state scans during each 60 minute functional scanning session. The inclusion of two pairs of resting-state scans was done in order to test for differences in cerebral blood flow (CBF) and resting state functional connectivity (RSFC) at different time-points post drug-administration. The first ASL scan began 50 minutes after oral administration of 100 mg encapsulated MDMA-HCl and on a separate occasion, placebo (encapsulated ascorbic acid/vitamin-C) and the second ASL scan began 103 minutes after capsule ingestion. The first resting-state BOLD scan took place 60 minutes after capsule ingestion and the second resting-state BOLD scan occurred 113 minutes after capsule ingestion (Figure 1). Participants relaxed with their eyes closed during the ASL and BOLD resting state scans. Peak subjective effects were reported 100 minutes post administration of MDMA, consistent with the plasma t-max of MDMA (2). The order of MDMA and placebo administration was counterbalanced. Behavioral paradigms not relevant to the present report were performed in the (~40 minute) period between the first and second pair of

ASL and BOLD resting-state scans. These included an autobiographical memory recollection and self-referencing task (note: the results of these paradigms will be reported separately).

Subjective Ratings

Participants gave ratings of the intensity of the subjective effects of MDMA using a simple visual analogue scale (VAS) completed via button press in the scanner. There was a bottom anchor of ‘no effects’ (or 0%) and a top anchor of ‘extremely intense effects’ (100%). The rating bar defaulted on ‘no effects’ and could be moved up in increments of 5% via button press (middle finger) and down in 5% increments via button press with the index finger. Subjects rated the intensity of the subjective effects before and after each resting state scan.

Participants also completed a more extensive list of VAS-style items to assess more specific subjective effects. This was completed 4.5 hours after capsule ingestion, when most of the subjective effects of MDMA had subsided. Some items were tailored to refer to commonly reported subjective effects of MDMA, expressed in colloquial terms (e.g. ‘I felt loved up’), and others were items used in previous research with psilocybin (3), selected in order to assist comparisons with the effects of this classic ‘psychedelic’ drug. The VAS scales had a bottom anchor of ‘no, not more than usually’ and a top anchor of ‘yes, much more than usually’ with the exception of a control item that read ‘I felt entirely normal’. In this case, the bottom anchor read ‘No, I experienced a different state altogether’ and the top anchor read ‘Yes, just as I usually feel’. There were 32 items in total in this questionnaire and its format was based on the APZ questionnaire for Altered States of Consciousness (4).

Cluster Analysis on Subjective Items

K-means clustering implemented in MATLAB was used to group the 29 subjective items (Figure 2) into 12 clusters. Four highly intercorrelated items were found that were specifically related to positive mood, namely: ‘I felt amazing’, ‘I felt loved-up’, ‘I felt a profound inner peace’,

'I felt an inner warmth'. A correlation matrix and dendogram confirmed the strong interrelatedness of these items (see Figure S1).

Correlation Analyses

Correlations between changes in CBF and RSFC and subjective ratings of effects intensity and positive affect were performed on data from anatomically defined regions of interest (ROIs). Pearson's *r* was used to calculate the statistical significance of correlations and statistical thresholds were corrected for multiple comparisons using Bonferroni correction. The mean for the four positive affect items were calculated for each subject and correlations were performed on the resultant positive mood ratings and specific changes in RSFC (expressed as beta values). Tests were corrected for multiple comparisons (.05/10 for the ASL analyses because correlations with 5 regions and 2 different subjective rating parameters were explored, and .05/6 for the RSFC analyses because 3 specific coupling parameters and 2 different subjective rating parameters were tested). For the positive mood correlations, all data points were included in the analyses; however, five participants gave ratings of zero for effects intensity while on MDMA in the scanner, despite reporting noticeable subjective effects on exiting it, and so these were considered null and removed. The ROIs for the analyses were defined using the Harvard/Oxford anatomical atlas available in the FMRIB Software Library (FSL: http://www.fmrib.ox.ac.uk/fsl).

ASL Analysis

The ASL time series for each inflow time scan was motion corrected using rigid body translation/rotation and then registered to each other using AFNI tools (http://afni.nimh.nih.gov/afni). For each inflow time scan, the tag and control time series were interpolated to the TR, subtracted and averaged. CBF and arterial arrival times were quantified by fitting a general kinetic model (5) to the resulting multi-inflow time data using a non-linear

fitting routine. The M0 of blood was estimated from the white matter signal in the M0 calibration scans assuming a ratio of the proton density of blood to that in white matter R = 1.06 (6). Other parameters used in the model were inflow cut-off time blood = 1.7, T*2 blood = 0.1 s (7), T*2 white-matter = 0.047 s (8) and q = 1 (6). The quantified CBF maps were registered to each individual's structural scan using transformations calculated from the original EPI data and were converted into standard Montreal Neurological Institute space using FLIRT within FSL. The free software 'MRIcron' was used to produce the images shown in Figure 3.

Functional Connectivity Analysis

FSL was used for the RSFC analyses. Three separate 'seed'-based functional connectivity analyses were performed using three a priori defined regions: the ventromedial prefrontal cortex (vmPFC), bilateral hippocampal and bilateral amygdala seeds. The vmPFC mask was the same as one previously used in an analysis involving psilocybin, to assist comparisons with this drug (3). The hippocampal mask was constructed by combining the right and left hippocampal structures and parahippocampal gyrus (anterior and posterior divisions) and thresholding this mask by 50% using the Harvard-Oxford probabilistic atlas. The same approach was taken for the bilateral amygdala mask, combining the left and right amygdala and thresholding the mask at 50%. Mean time series were derived from these masks for each participant's 4 scans (i.e. placebo rest 1 and 2 and MDMA rest 1 and 2) and the 'fsl_motion_outliers' command was used to identify outlier volumes that could then be entered as confound variables in the relevant regression analyses to further control for between-condition differences in motion. Time series from spheres placed in the white matter (WM) and cerebrospinal fluid (CSF) were acquired, as was the average gray matter (GM) signal from a whole brain GM mask. These data were included as explanatory variables of no-interest, to control for these potential confounds.

General linear modeling (GLM) was used to assess between-condition (i.e. drug versus placebo) differences in functional connectivity. At the single subject level, a high-pass filter of 100 seconds was used, each volume was spatially smoothed (5 mm full-width half-maximum) and motion regressors were added to the model. Each model contained 5 explanatory variables: the time series from the ROI or seed (i.e., the vmPFC, hippocampus or amygdala) plus the GM, WM and CSF time series, plus a text file indicating the temporal occurrence of outlier volumes as an additional confound variable. The first temporal derivative was included in the GLM and first level contrasts were set to detect effects in clusters of voxels where there was positive coupling to the ROI. The functional images were registered to each individual's T1 anatomical scan and then to a standard brain.

For the higher-level analyses, maps were produced to reveal locations of significant coupling to each ROI (see Figure S5). Each subject's two placebo resting state scans were included in the GLM and a mixed-effects ordinary least squares analysis was run to produce maps using a default cluster threshold of $Z > 2.3$, $p < 0.05$. To compare the MDMA and placebo conditions, all of the subject's scans were included as inputs to the model and the MDMA condition was contrasted with the placebo condition using the same statistical thresholds as above. Of potentially 100 resting-state scans, only 2 could not be analyzed due to extreme movement (one under placebo and one MDMA). MRIcron was used to produce the displayed maps.

Addressing Potential Confounds

With the aim of strengthening the inferences that could be made on the study outcomes, a number of additional regression analyses were performed to address the influence of potential confounds. Specifically, contrast images reflecting the difference in RSFC under MDMA versus placebo were entered into additional GLM analyses with mean motion per-scan entered as an explanatory variable. If no relationships could be found between these variables, then it would

imply that movement had not caused the between-condition differences in RSFC. The same procedure was used to test if lifetime exposure to and recency of MDMA-use, as well as lifetime cannabis-use and weekly alcohol-use could explain the between-condition differences in RSFC. If they could not, then these potential confounds could be ruled out as causes of the differences in RSFC. Each variable was tested separately for all 3 seed-based RSFC analyses, totaling 12 different tests.

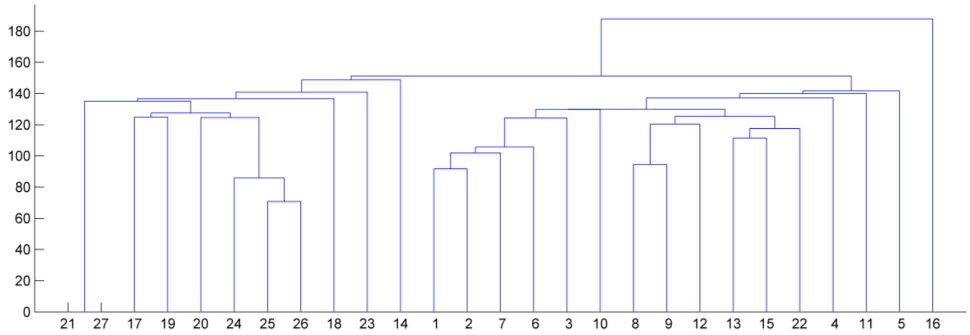

Figure S1. Dendogram. K-means clustering was used to parcelate the 29 subjective items (Figure 2) into 12 clusters. Items 1, 2, 7 and 6 are intercorrelated. These were: 1) 'I felt amazing', 2) 'I felt an inner warmth', 6) 'I felt a profound inner peace' and 7) 'I felt loved-up'.

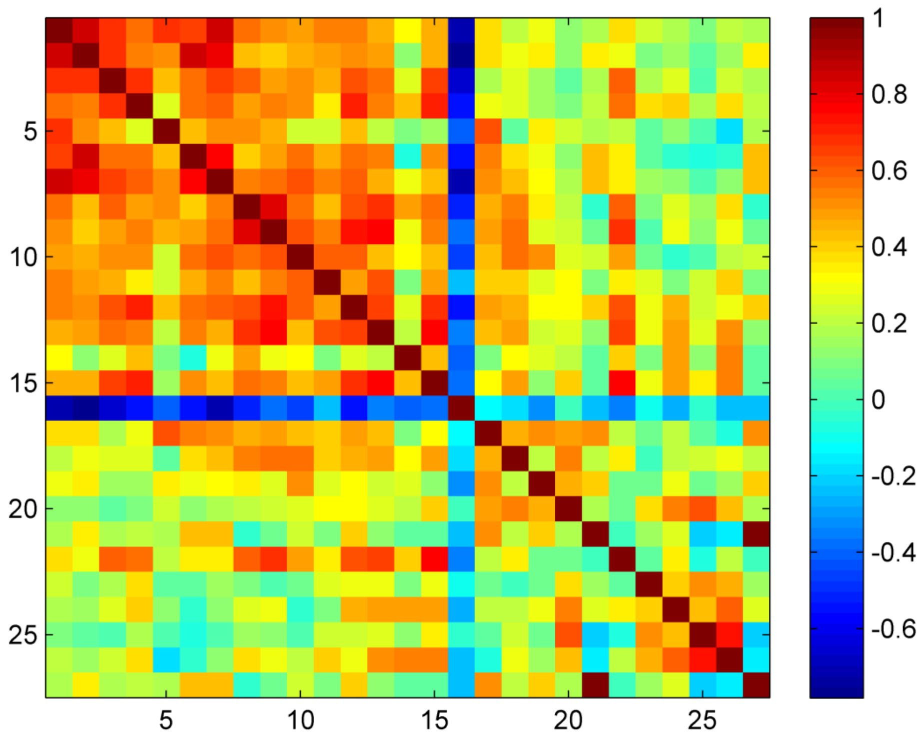

Figure S2. Correlation matrix. Items (1-29) are stacked as in Figure 2 of the main paper. It is evident that items 1, 2, 6 and 7 are highly intercorrelated. Colors reflect the strength of the pair-wise correlations.

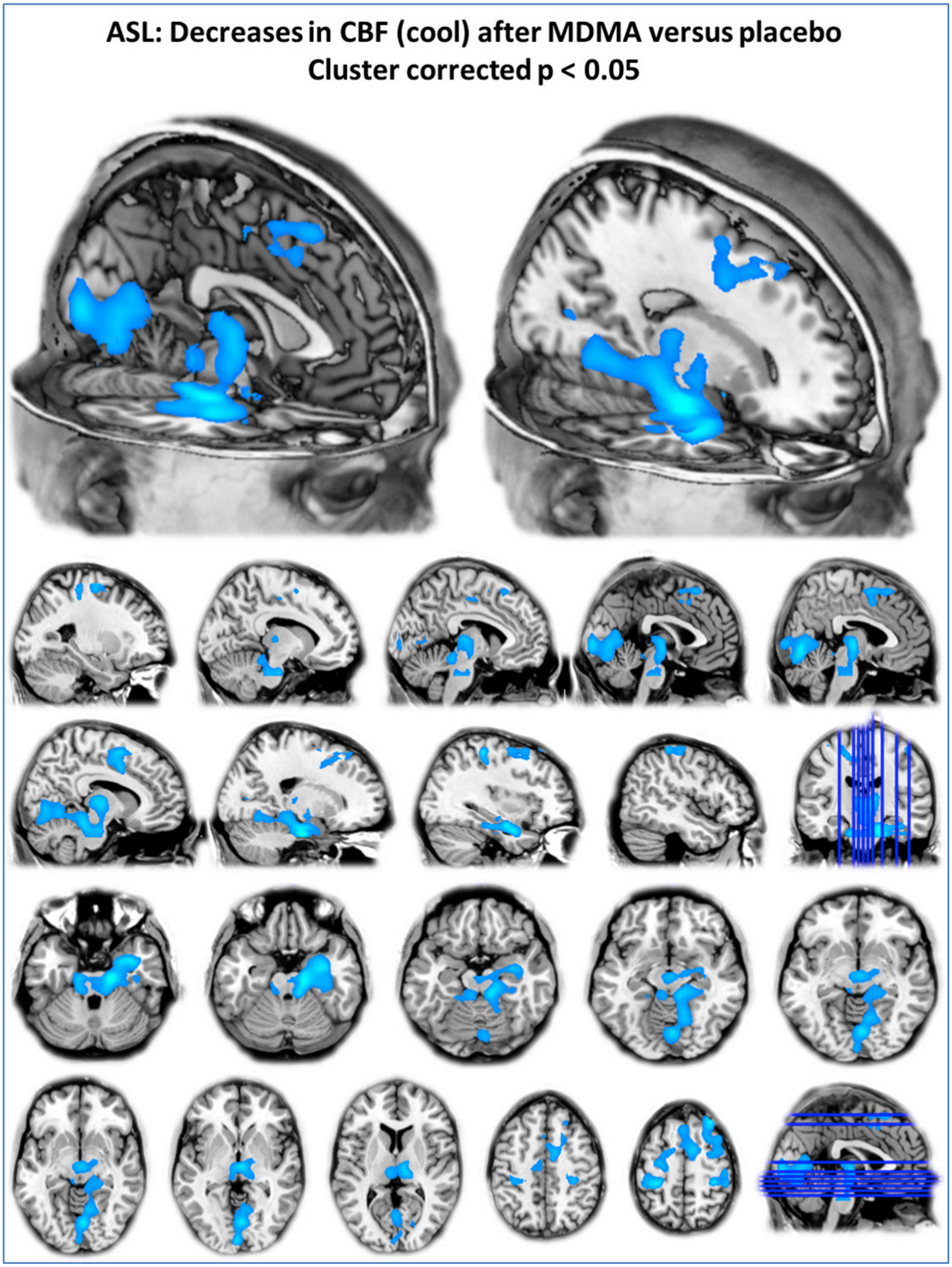

Figure S3. More slices showing changes in CBF post-MDMA v post-placebo. Cluster corrected, $p < .05$, $n = 25$. ASL, arterial spin labeling; CBF, cerebral blood flow.

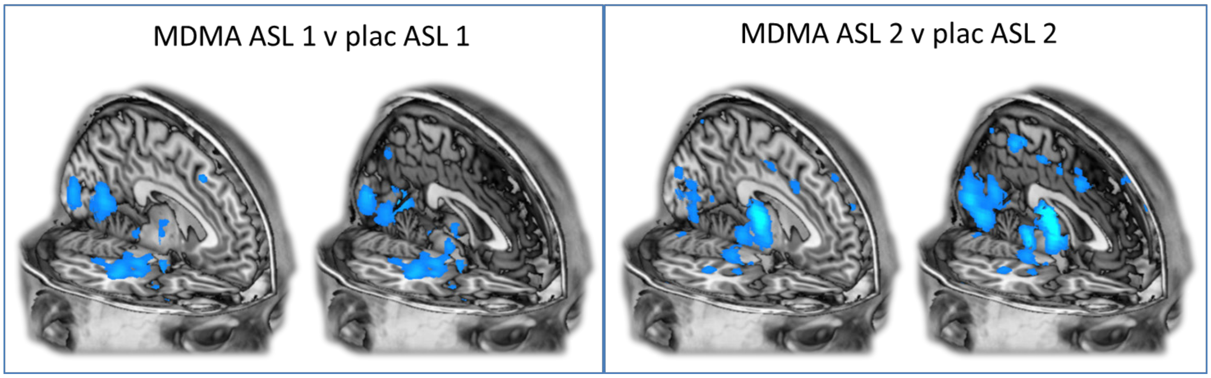

Figure S4. The changes in CBF (MDMA v placebo) in the first ASL scan (left) and the second ASL scan (right). The default statistical threshold was used, i.e. cluster-corrected, $p < .05$. ASL, arterial spin labeling; CBF, cerebral blood flow; plac, placebo.

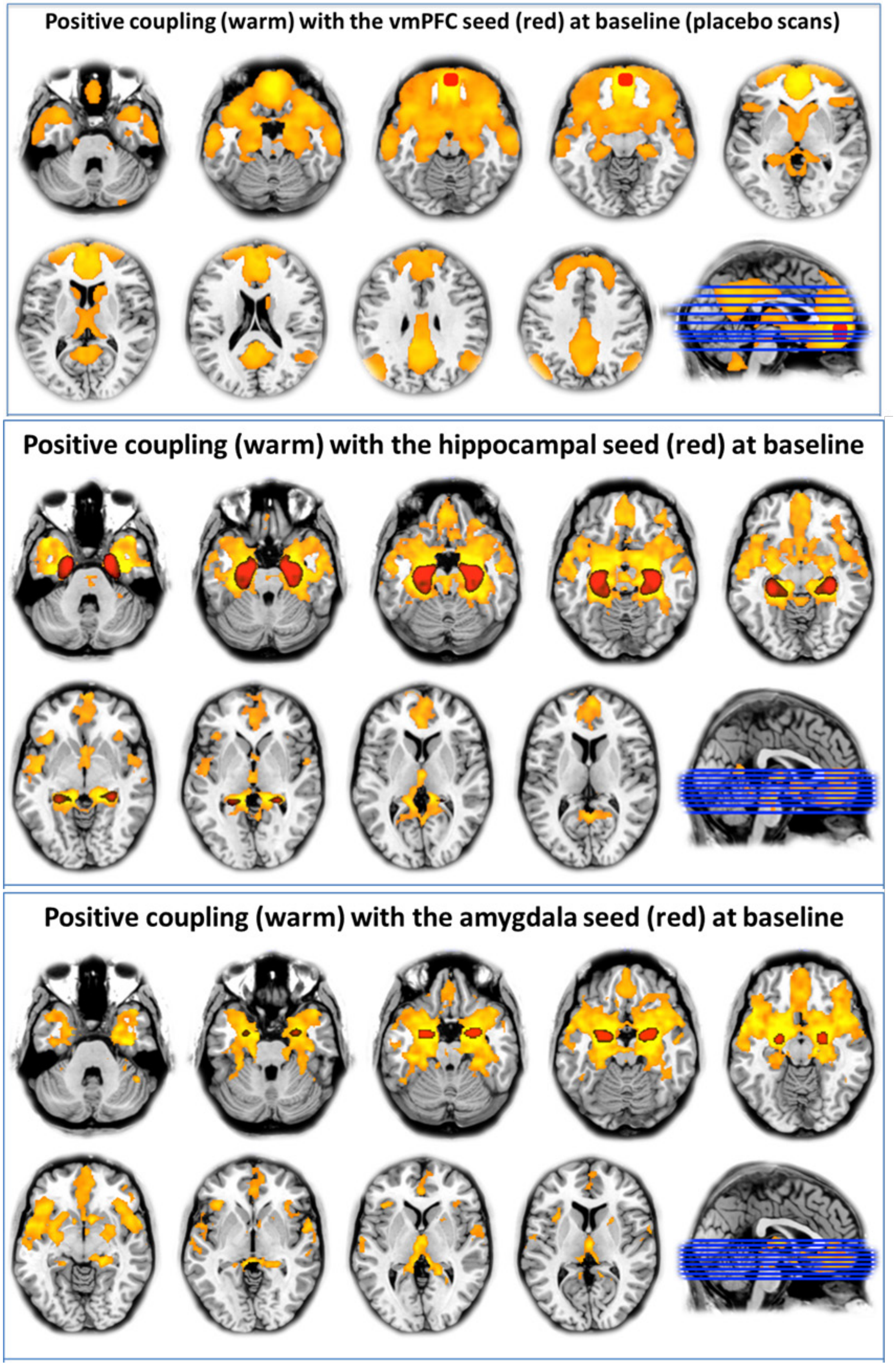

Figure S5. Positive RSFC for the 3 ROIs used in the seed-based RSFC analyses reported in Figure 4 of the main paper. Cluster-correction, z = 2.3, *p* < .05. ROIs, regions of interest; RSFC, state functional connectivity; vmPFC, ventromedial prefrontal cortex.

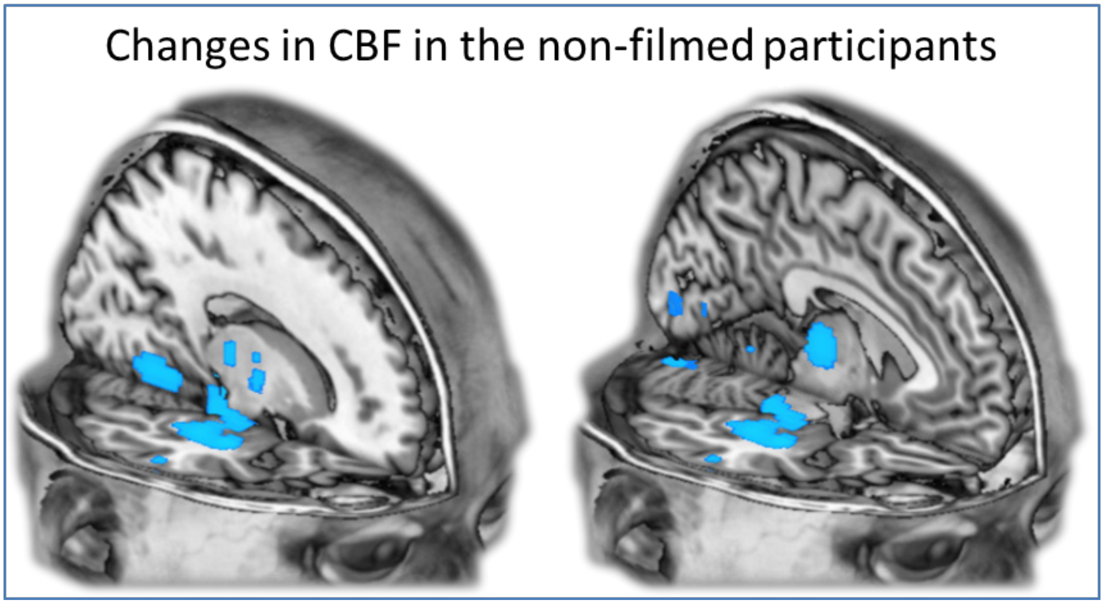

Figure S6. Changes in CBF post-MDMA vs post-placebo in the non-filmed subjects. Cluster corrected, $p < .05$, $n = 20$. Note that the main effect of MDMA is still a decrease in CBF and in consistent regions to the main sample of 25. CBF, cerebral blood flow.

Supplemental References

1. Varnas K, Nyberg S, Halldin C, Varrone A, Takano A, Karlsson P, *et al.* (2011): Quantitative analysis of [11C]AZ10419369 binding to 5-HT1B receptors in human brain. *J Cereb Blood Flow Metab.* 31:113-123.
2. Wilson MA, Molliver ME (1991): The organization of serotonergic projections to cerebral cortex in primates: regional distribution of axon terminals. *Neuroscience*. 44:537-553.
3. Carhart-Harris RL, Erritzoe D, Williams T, Stone JM, Reed LJ, Colasanti A, *et al.* (2012): Neural correlates of the psychedelic state as determined by fMRI studies with psilocybin. *Proc Natl Acad Sci U S A.* 109:2138-2143.
4. Reader TA, Dewar KM, Grondin L (1989): Distribution of monoamines and metabolites in rabbit neostriatum, hippocampus and cortex. *Brain Res Bull.* 23:237-247
5. Kohler C, Radesater AC, Lang W, Chan-Palay V (1986): Distribution of serotonin-1A receptors in the monkey and the postmortem human hippocampal region. A quantitative autoradiographic study using the selective agonist [3H]8-OH-DPAT. *Neurosci Lett.* 72:43-48.
6. Costes N, Merlet I, Ostrowsky K, Faillenot I, Lavenne F, Zimmer L, *et al.* (2005): A 18F-MPPF PET normative database of 5-HT1A receptor binding in men and women over aging. *J Nucl Med.* 46:1980-1989.
7. Andrade R (2011): Serotonergic regulation of neuronal excitability in the prefrontal cortex. *Neuropharmacology*. 61:382-386.
8. Hoyer D, Pazos A, Probst A, Palacios JM (1986): Serotonin receptors in the human brain. II. Characterization and autoradiographic localization of 5-HT1C and 5-HT2 recognition sites. *Brain Res*. 376:97-107.
